# An Italian-Mediterranean Dietary Pattern Developed Based on the EAT-Lancet Reference Diet (EAT-IT): A Nutritional Evaluation

**DOI:** 10.3390/foods10030558

**Published:** 2021-03-08

**Authors:** Massimiliano Tucci, Daniela Martini, Cristian Del Bo’, Mirko Marino, Alberto Battezzati, Simona Bertoli, Marisa Porrini, Patrizia Riso

**Affiliations:** 1 Department of Food, Environmental and Nutritional Sciences (DeFENS), Università degli Studi di Milano, 20133 Milan, Italy; massimiliano.tucci@unimi.it (M.T.); daniela.martini@unimi.it (D.M.); cristian.delbo@unimi.it (C.D.B.); mirko.marino@unimi.it (M.M.); alberto.battezzati@unimi.it (A.B.); simona.bertoli@unimi.it (S.B.); marisa.porrini@unimi.it (M.P.); 2 International Center for the Assessment of Nutritional Status (ICANS), Università degli Studi di Milano, 20133 Milan, Italy

**Keywords:** healthy and sustainable diet, planetary healthy diet, nutrition, sustainability, nutritional adequacy, environmental impact, Mediterranean diet, dietary guidelines

## Abstract

There is an urgent need to promote healthy and sustainable diets that are tailored to the preferences and cultures of different populations. The present study aimed to (i) define a Mediterranean dietary pattern in line with the EAT-Lancet Commission reference diet (ELCRD), based on 2500 kcal/day and adapted to the Italian food habits (EAT-IT); (ii) develop a mid/long-term dietary plan based on EAT-IT and a dietary plan based on the Italian Dietary Guidelines (IDG); (iii) compare the two dietary plans in terms of portions, frequencies of consumption, and nutritional adequacy based on the nutrient and energy recommendations for the Italian adult population. The main differences between the two plans were related to the higher amount of fruit and vegetables in the IDG compared to the EAT-IT, while the EAT-IT plan was higher in nuts and legumes, which represent the main protein sources in the ELCRD. Differences in the protein sources, especially milk and derivatives, and for cereal-based foods, were also found. Dietary plans were comparable for most nutrients, except for higher energy from lipids and vegetal protein, a higher amount of fiber, and lower levels of calcium that were evidenced for the EAT-IT dietary plan compared to the IDG-based one. In conclusion, the analysis of the EAT-IT demonstrated certain nutritional issues. It remains to be determined whether this may represent a health concern in further studies aimed at investigating the feasibility of sustainable dietary patterns.

## 1. Introduction

A large body of evidence demonstrates the role of poor diet on malnutrition and the potential impact of a suboptimal diet on mortality and morbidity for noncommunicable diseases. The Global Burden of Disease Study 2017 estimated that, in the adult population (older than 25 years), 22% of total deaths (11 million total) and 15% of disability-adjusted life-years (255 million total) across 195 different countries are attributable to dietary risks, such as the low intake of whole grain, fruits, nuts, vegetables, and omega-3 fatty acids and the excessive intake of sodium [1].

In addition to the effect of a poor diet on human health, the growing degradation of natural resources led to an increased interest in evaluating the impact of food choices (and today’s dietary guidelines) on planetary health [2,3,4]. Food systems indeed account for a large part of land and water use and of greenhouse gas emissions due to agriculture but also to processing, packaging, refrigeration, transport, retail, catering, domestic food management, and waste disposal (landfills) [4,5,6].

In this scenario, there is an urgent need to promote healthy diets that at the same time have a low environmental impact, are socioculturally acceptable, and are economically accessible, as highlighted by the Food and Agriculture Organization of the United Nations (FAO), which defines sustainable diets as “those diets with low environmental impacts which contribute to food and nutrition security and to healthy life for present and future generations. Sustainable diets are protective and respectful of biodiversity and ecosystems, culturally acceptable, accessible, economically fair and affordable; nutritionally adequate, safe and healthy; while optimizing natural and human resources” [7]. A large body of evidence suggests moving toward dietary plant-based diets that are effective in improving human health, and at the same time, in reducing environmental impacts [8]. However, it is noteworthy that this does not necessarily mean a shift to vegetarian and/or vegan diets, which some studies have shown to be nutritionally inadequate [9] and not always related to a lower ecological footprint compared to other diets that include modest amounts of animal-based foods [10].

The report of the EAT-Lancet Commission on healthy diets from sustainable food systems [11] aimed to establish global targets that are useful for defining a safe operating space for food systems, enabling them to assess which diets and food production practices can help to ensure that the United Nations (UN) Sustainable Development Goals [12] and Paris Agreement are achieved. The EAT-Lancet Commission Reference Diet (ELCRD) (see Appendix A) is mainly characterized by whole grains, vegetables, legumes, nuts, unsaturated oils, low amounts of seafood and poultry, and low or no red meat, processed meat, added sugar, refined grains, or starchy vegetables. Research has estimated that a shift from the current global diet to this healthy diet could prevent ≈24% of the total deaths from 2017 [13].

A peculiarity of this report is the description of a universal healthy reference diet that was developed with the aim to provide a basis for estimating the health and environmental effects of adopting an alternative diet relative to the standard current diets. According to the Commission, this pattern allows for the flexible, global application of specific criteria within a safe operating space, with foods and amounts tailored to the preferences and cultures of different populations. For this reason, it has been referred to as a “planetary diet” since it can and should be adapted to develop meals that are consistent with food cultures and cuisines of the different countries, maintaining both healthiness and environmental sustainability [11].

Previous studies have developed applications of the ELCRD with the specific purpose to compare different dietary patterns. In detail, Lassen et al. [14] proposed a culturally adapted ELCRD for the Denmark population through the adjustment of the energy target and portion size of different food categories to increase compliance with the Danish dietary guidelines. Sharma et al. [15] compared the ELCRD indications for the food intake of rural and urban households, as well as poor and rich households, of different Indian regions, highlighting critical points and identifying actions to orientate policies. Differently, Blackstone and Conrad [16] investigated how this pattern differs from American national guidelines, which currently do not include adjustments related to environmental sustainability. The authors highlighted that, despite some similarities between the EAT-Lancet and American dietary guidelines, where the latter recommend higher amounts of fruit, starchy vegetables, red meat, and discretionary calories and lower amounts of nuts, seeds, and whole grains compared to the ELCRD. 

To the best of our knowledge, no similar studies have been performed in Mediterranean countries, where, as reported by the EAT-Lancet Commission, the typical dietary pattern, i.e., the Mediterranean diet, has some features in common with the ELCRD, being a dietary plant-based diet with low red meat but high total fat intake mainly due to olive oil [17]. Based on these premises, the present work pursued the following three objectives: (i) to develop a Mediterranean-based dietary pattern in line with the EAT-Lancet Commission reference diet, which was adapted by considering the Italian food habits and culture; (ii) to translate this dietary pattern into an example of a feasible and sustainable mid/long-term dietary plan that is able to cover nutrient requirements; (iii) to develop a similar dietary plan that is in line with the Italian Dietary Guidelines (IDG) [18]; (iv) to compare the two dietary plans in terms of portions, frequencies of consumption, and overall nutritional adequacy for the Italian adult population.

## 2. Materials and Methods

**Figure 1:**
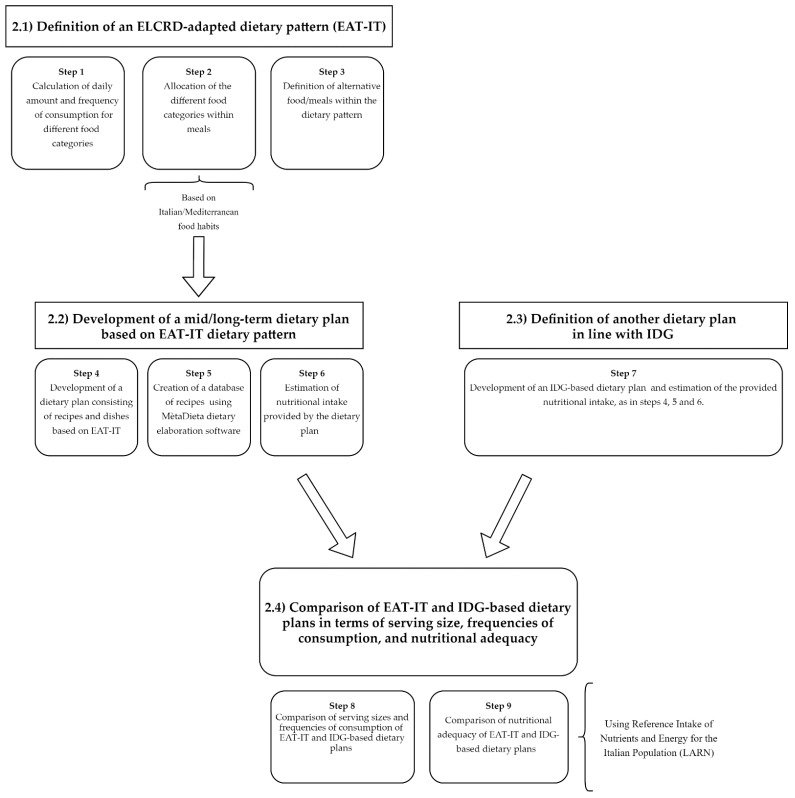
Overview of the protocol used for the development and analysis of the dietary pattern based on the EAT-Lancet Commission Reference Diet (ELCRD) that was adapted by considering the Mediterranean/Italian food habits (EAT-IT). IDG: Italian dietary Guidelines.

The main steps that were taken to adapt the ELRCD-based dietary plan to Italian eating habits and to compare it with an IDG-based dietary plan are shown in Figure 1 and described in detail below. Overall, the process was divided into four main phases:

- Definition of the ELCRD adapted dietary pattern (named EAT-IT).
- Development of a mid/long-term dietary plan based on the EAT-IT dietary pattern.
- Definition of another dietary plan in line with IDG, which was useful as a basis for comparing nutritional intakes.
- Comparison of the EAT-IT-based and IDG-based dietary plans in terms of serving size, frequencies of consumption, and nutritional adequacy.

### 2.1. Phase 1: Definition of the EAT-IT-Based Dietary Plan

This first phase consisted of three steps that were devoted to the definition of an ELCRD-adapted dietary pattern based on Mediterranean food habits. The ELCRD provides the daily intake for eight different food categories (whole grain, tubers or starchy vegetables, vegetables, fruits, dairy foods, protein sources, added fats, and added sugars), which are expressed as both grams/day and kilocalories/day considering a diet of 2500 kcal/day [11], while no information regarding the frequencies of consumption or other indications related to the meal preparation are provided. For these reasons, with the aim to develop a mid/long-term dietary pattern, the daily intakes were converted to weekly amounts (step 1) that were expressed as grams. Data were used to calculate feasible weekly frequencies of consumption, while the intake expressed as kilocalories was used to develop isocaloric alternatives within the same food category. Once we calculated the portion and the frequency of consumption for the eight categories, foods were allocated into different meals (step 2). This was performed in line with the indications from the Mediterranean food pyramid, which, for instance, includes cereal-based products and fruits and vegetables at every main meal. 

The final step (step 3) consisted of the development of a dietary pattern, putting together the meals designed in step 2 but providing alternatives that would allow for following this dietary pattern for the long term. The resulting scheme was named EAT-IT, as it was rearranged based on the Italian/Mediterranean food habits and in line with the indications from the EAT-Lancet Commission. 

### 2.2. Phase 2: Simulation of an EAT-IT-Based Dietary Plan

The second phase included three steps that were devoted to the development of a dietary plan, which was based on the dietary pattern planned as described in Section 2.1 and considering 2500 kcal/day as the target energy intake. First, recipes and dishes, based on traditional and generally consumed recipes that are eaten by the Italian population, were constructed based on the EAT-IT scheme and considering an adequate number of alternatives/substitutions with the purpose to cover a mid-to-long period (i.e., several months/a season) (step 4). For instance, for protein sources, several recipes were used to offer an adequate number of alternatives, with the aim to increase the variability of the dietary plan and to simulate a situation in which the consumers can choose different dishes based on their food preferences and ingredient availability. The number of alternatives proposed was greater for food categories with higher frequencies of consumption in traditional Italian meals.

These recipes were then used to create a database that was elaborated upon using software for nutritional assessment (MètaDieta professional 4.1.1 METEDA Srl–Roma, Italy) to simulate a monthly dietary plan that is consistent with Italian habits (step 5). Specifically, five meals per day were considered (breakfast, lunch, dinner, and mid-morning and mid-afternoon snacks), including a “typical” Italian breakfast [19] made of a cereal-based food, a dairy product, and jam or juice, as well as snacks [20] consisting of nuts.

Once developed, the dietary plan was analyzed for the nutritional characteristics in terms of energy and macro- and micronutrients, and finally provided as daily amounts in kilocalories, grams, milligrams, or micrograms, depending on the nutritional component (step 6).

### 2.3. Phase 3: Simulation of a Dietary Plan in Line with IDG

This third phase of the process was aimed at defining a dietary plan that was developed based on the IDG, similar to what was performed in Section 2.2. Toward this aim, in step 7, we performed an elaboration of a mid/long-term dietary plan following the same procedure that was used for elaborating the EAT-IT plan. 

### 2.4. Phase 4: Comparison of the Portions, Frequencies of Consumption, and Nutritional Adequacy of the EAT-IT- and IDG-Based Dietary Plans

The final phase of this process was devoted to the comparison of the newly developed IDG-based dietary plan with the EAT-IT plan in terms of the serving sizes, frequencies, and nutritional adequacy. Specifically, in step 8, the EAT-IT serving sizes and frequencies of consumption were compared with those proposed in the last edition of the IDG and referring to the target energy intake of 2500 kcal. Finally, the nutritional adequacy of the two developed dietary plans was analyzed (step 9). The comparison was made by considering the nutritional adequacy of the two dietary plans, i.e., their ability to reach the target intakes and to cover the reference values for energy and macro- and micronutrients as defined for Italian adults with 2500 kcal as the recommended energy intake (both men and women), using the Levels of Reference Intake of Nutrients and Energy for the Italian Population (LARN) as a reference [21]. 

In detail, data were compared with reference intakes (RIs, for carbohydrates and lipids). The average requirements (ARs) and the population reference intake (PRI) were instead used when available (i.e., for protein, all vitamins except vitamin E, and all minerals except sodium, chlorine, and potassium). The AR represents the level of nutrient intake sufficient to satisfy the needs of 50% of healthy subjects of a specific group of a population while the PRI refers to the amount needed to cover 97.5% of the population, thus providing important information for nutritional assessment. For the micronutrients for which AR was not available, adequate intake (AI) was considered (vitamin E, sodium, chlorine, and potassium). When available (i.e., for saturated fatty acids (SFA), cholesterol, sugar, and fiber), the suggested dietary target (SDT) was also taken into account for nutritional evaluation. 

## 3. Results

### 3.1. Definition of the EAT-IT Dietary Pattern and Simulation of a Consistent Dietary Plan

As described in the Materials and Methods section (see Section 2.1), step 1 was devoted to the calculation of the daily or weekly servings for the different foods based on the ELCRD model. These portions, expressed as grams/week, were then used to calculate feasible weekly frequencies of consumption. For instance, for the subcategory “eggs” (with a daily intake of 13 g/day based on the ELCRD), 91 g/week were calculated, which corresponded to about two eggs of 50 g per week. In addition, the food portions were expressed in kilocalories/week to develop isocaloric alternatives within the same food category: for instance, 250 mL of whole milk was considered equivalent to 330 mL of semi-skimmed milk. 

In step 2, the different foods were allocated in the daily meals (i.e., breakfast, lunch, dinner, and snacks) according to the Mediterranean food pyramid [17], as summarized in Table 1 and Table 2, in which meal compositions and possible alternatives are reported. By using combinations of such meals, the EAT-IT dietary pattern was developed (step 3) to be adherent to the ELCRD indication but adapted to the Italian dietary habits and recipes, and sufficiently varied to be followed for a relatively long period. In detail, the single meals were organized as follows: (1) breakfast included a source of whole grains (e.g., oat flakes or wholemeal rusks), milk or derivatives, and added sugars (e.g., jam or fruit juice); (2) lunch and dinner were composed of a source of whole grains (e.g., brown rice, corn, or wholemeal pasta) or starchy vegetables (i.e., potatoes), a protein source (e.g., legumes, chicken and poultry, beef, lamb and pork, or fish), vegetables, fats (mainly extra virgin olive oil), and a portion of fruit at the end of each meal; (3) snacks (twice a day) consisting of a portion of nuts.

Once we defined the EAT-IT dietary pattern, a complete dietary plan with recipes and alternatives was developed to simulate a real-life diet with meals and preparations that were chosen to include all foods in the defined servings and frequencies but remaining consistent with the Italian culinary tradition (steps 4 and 5). For example, to facilitate the use of large amounts of legumes, traditional recipes already present in Italian food habits, such as “pasta e fagioli” (i.e., pasta with “borlotti” beans) or “riso e bisi” (rice with peas), were included (data not shown). The newly developed dietary plan was finally evaluated in terms of the nutritional characteristics, as described below (Section 3.3). 

### 3.2. Comparison of the Serving Sizes and Frequencies of Consumption between the Italian Dietary Guidelines and the EAT-IT Dietary Patterns

As described in Section 2.4, the indications provided by the last edition of the Italian Dietary Guidelines [18] in terms of both serving sizes and frequencies of consumption were used to develop a parallel dietary plan (step 7, data not shown). This IDG-based dietary plan was compared with the EAT-IT dietary plan, as reported in Table 3. 

Interestingly, one of the main differences between the two dietary patterns was related to the amount of fruits and vegetables provided, which was lower in the EAT-IT compared to the IDG dietary plans (overall 200 g/day vs. 450 g/day for fresh fruits and overall 300 g/day vs. up to 600 g/day of fresh vegetables, respectively). Large differences were also observed for protein sources, with a lower amount of chicken meat in the EAT-IT, compared to the IDG (overall 200 g/week vs. 300 g/week), as well as fish (200 g/week vs. 300 g/week), eggs (four medium eggs of 50 g/week vs. two medium eggs), and milk and derivatives (overall 375 mL/day of milk or yogurt vs. 250 mL/day). The Italian guidelines suggest three portions of cheese/week, while cheese is only included as an alternative for milk in the EAT-IT. 

Conversely, compared to the IDG, the EAT-IT provided a higher quantity of legumes (eight portions/week vs. three portions of 150 g of fresh legumes or 50 g of dried legumes/week) and nuts (45 g day vs. 2.5 portions of 30 g), while red meat was comparable (100 g/week). The comparison for “cereals and derivatives” was tricky since the indications are different in the two schemes, for example, with the Italian guidelines suggesting 4.5 portions of bread/day (for a total of 225 g/day) and one portion/day of 120 g of pasta, rice, corn, and other cereals, while the EAT-IT potentially allowed for bread up to a maximum of 300 g/day or cereals twice a day (for a maximum daily amount of 190 g). 

The IDG also includes indications related to the intake of sweet bakery products, which are not specifically included in the EAT-IT dietary pattern. Therefore, a comparison was not easy due to the substantial differences in the nature of the indications provided for this category. Finally, an important difference between the two dietary plans was related to further indications provided by the IDG that were not present in the EAT-IT, e.g., suggestions of equivalences for dried fruit and portion and frequencies of consumption for leaf salads, preserved fish, and water. 

### 3.3. Assessment of the Nutritional Adequacy of the EAT-IT- and IDG-Based Dietary Plans

In this last part of the process, the energies and nutrient intakes of the two developed dietary plans (i.e., the EAT-IT- and IDG-based dietary plans) were analyzed to assess their nutritional characteristics. In detail, the energy and macro- and micronutrients provided by the two dietary plans were compared with the Italian recommendations (LARN [21]) developed by the Italian Society of Human Nutrition (SINU) to verify the ability of these dietary patterns to satisfy the nutritional requirements, considering an energy target of 2500 kcal. 

Table 4 shows the comparison of the macronutrient composition and distribution of the two dietary patterns. The overall higher amount of lipids provided by the EAT-IT dietary plan (36.2%) compared to the IDG-based dietary plan accounted for 30.3% of the daily energy on average. The difference in the lipids intake appeared to be mainly due to the monounsaturated fatty acids (MUFA), which were considerably higher in the EAT-IT-based dietary plan (19.6%) relative to the IDG plan (14.9%). The high amount of lipids in the EAT-IT plan was accompanied by a relatively low amount of carbohydrates (about 48%), which was slightly above the lower threshold set at 45% of energy by LARN. In the IDG dietary plan, carbohydrates provided about 54% of energy (including energy from fiber). 

Regarding the total fiber, the amount was higher in the EAT-IT dietary plan relative to the IDG and slightly higher than the range reported in the LARN (12.6–16.7 g/1000 kcal). Despite these differences, both dietary plans were verified to cover the requirements for the majority of the nutrients, including some of the most critical ones, such as omega-3 essential fatty acids (0.6% and 0.8% of energy for the IDG and EAT-IT, respectively) and protein was comparable between the two dietary patterns (97.6 and 97.7 g, respectively). Energy from omega-6 fatty acids was within the range (RI = 4–8% of energy) for the EAT-IT plan (6.3%) and just slightly below the RI for the IDG (3.7%). Notably, both dietary plans provided low amounts of cholesterol (less than the 300 mg/day suggested by the LARN), where the cholesterol level was markedly lower in the EAT-IT-based dietary plan (165.4 mg) compared with the IDG plan (248.7 mg). 

Considering vitamins (Table 5), no nutritional inadequacy emerged from the analysis of the two dietary plans, except for vitamin D, which was found to be lower than the average requirement indicated by the LARN (10 µg) in both the IDG and EAT-IT dietary plans (2.3 and 1.9 µg, respectively).

For other vitamins, such as vitamin K (phylloquinone and menaquinone), B5 (pantothenic acid), and B8 (biotin), a thorough evaluation could not be carried out due to the presence of many missing data for these vitamins in the food composition databases used (i.e., the Food Composition Database for Epidemiological Studies in Italy [23] and the Council for Agricultural Research and Economics (CREA) food composition database [24]).

Regarding the mineral intake, the comparison of the dietary plans revealed wide differences in the calcium levels provided by the two dietary plans. Specifically, the EAT-IT-based dietary plan was less prone to satisfy the average requirement defined by the LARN, providing, on average, 680 mg/day (Table 6). The overall iron was found to be adequate in both the IDG- (17.9 mg) and EAT-IT-based dietary plans (22.1 mg), even when compared to the PRI (18 mg for female and 10 mg for male) reported by the LARN, and with the higher amount provided by the EAT-IT (≈22 mg), despite the overall lower amount of meat (considering both white and red meat) when compared to the IDG. 

All other assessed minerals (magnesium, phosphorus, potassium, and zinc) were adequate in both dietary plans. Sodium was found to be lower (about 827 mg) in the EAT-IT dietary plan compared to the AI suggested by the LARN (1500 mg) and close to the SDT (2000 mg) for IDG (≈2017 mg), while chlorine was found to be lower than the AI (2300 mg) in both the IDG- and EAT-IT-based dietary plans (1217 and 531 mg, respectively). Regarding the values of sodium and chlorine, discretionary salt was not included in the evaluation. As already reported for vitamins, the adequacy of certain minerals, such as copper, iodine, manganese, and selenium, was not assessed due to missing data in the database.

## 4. Discussion

In this study, we developed a practical application of the EAT-Lancet Reference Diet into a dietary plan that is consistent with Italian/Mediterranean food habits. Indeed, the sustainability of diets represents a crucial issue for the future [25], as healthy and sustainable diets should be both adequate for satisfying nutritional requirements and respectful of local traditions and cultures [7]. In this context, the last revision of the IDG [18], which was developed by the CREA Food and Nutrition Research Centre, has provided dedicated information on this specific issue (i.e., “How to ensure a varied, safe, healthy and sustainable diet”), highlighting how an adequate consumption of the different food groups of the Italian tradition, including limited amounts of animal products, can positively impact both on humans’ and the planet’s health. 

The planetary diet is based on whole grains, legumes, nuts, fruit, and vegetables and includes a limited amount of dairy, meat, and other animal sources of protein and fats. The current literature is certainly not complete, but diets including reduced amounts of meat and dairy are indicated by many studies as both having a lower impact on the environment and being nutritionally adequate [9,26]. 

Collins and Fairchild [10] calculated that, based on the food consumption of the Cardiff population, the lowest environmental impact was obtained with a partial substitution of food with a high ecological footprint, while a typical vegetarian diet was associated with a lower reduction in the ecological footprint and lower nutritional adequacy. Seves et al. [9] performed similar calculations based on the food consumption of the Dutch population, reporting that a vegan diet (all meat and dairy replaced with plant-based foods) was associated with the highest reduction in greenhouse gas emissions and land use but was nutritionally inadequate, while a 30% replacement, despite having a less marked reduction on the environmental impact, resulted in the best performance in terms of nutritional adequacy. These considerations underline the importance of better evaluating the pros and cons of modifications in the traditional diet and the need for a better understanding of the possible nutritional and functional impacts of revised, sustainable dietary patterns. 

The adaptability and scalability of the ELCRD have been investigated from different points of view in studies performed in different countries, including the USA [16], India [15], and Denmark [14]. Considering the Italian scenario, Ferrari et al. [27] calculated the daily portion of foods that could minimize gas emissions in an optimized Italian diet, while maintaining, as much as possible, an adequate nutritional intake. Conversely, no studies were found that previously developed a Mediterranean-Italian dietary pattern based on the EAT-Lancet reference diet.

We performed a nutritional adequacy assessment on two examples of dietary plans (one from the EAT-IT dietary pattern and one based on the IDG), which highlighted some potential issues related to the frequency of consumption of some foods/food classes and/or the intake of specific nutrients. For instance, the differences in the levels of energy from carbohydrates reflected slight differences in the serving sizes of cereals, particularly during breakfast. Indeed, the IDG includes specific recommendations regarding the consumption of biscuits, pastries, or other cereal-based products at breakfast, making this meal rich in carbohydrates sources, while the EAT-IT pattern includes a lower amount of wholemeal cereals. 

Conversely, the high amount of energy from lipids and the high amount of fiber in the EAT-IT dietary plan likely reflected the large intake of plant oil, nuts, and legumes, which represented the main differences between the two dietary patterns analyzed. Legumes and nuts are used in EAT-IT as an alternative for other protein sources, as reflected by the lower amount of total meat, fish, eggs, and dairy in the EAT-IT compared to the IDG. 

Regarding the nutritional adequacy of the two developed dietary plans, there was a higher fat intake promoted through the EAT-IT diet compared to the IDG one, where this was slightly higher than the LARN reference intake (i.e., 20–35% En). Despite being quite high, this value does not exceed the SDT for SFA and mainly consisted of MUFA because extra virgin olive was the primary source of fat in both dietary plans. An extensive review of randomized controlled trials demonstrated that dietary MUFA (20–25% of the total energy) prevented or ameliorated cardiovascular disease by modulating several biological parameters, such as the lipids profile, blood pressure, and insulin sensitivity [28]. The EAT-IT-based dietary plan also resulted in higher PUFA compared to the IDG dietary plan due to the higher amount of nuts. 

Other differences were observed for micronutrients. The level of calcium intake apparently provided by the EAT-IT dietary plan (about 675 mg) was low relative to the IDG plan (>1 g) and compared to the average requirements for adults (i.e., 800 mg/day). The intake of calcium represents a critical issue, considering that the mean daily intake from food in adults, according to the last available Italian National Food Consumption Survey INRAN-SCAI 2005-06 [29], is already lower than recommended (i.e., the average intake estimated: 756 mg for males and 697 mg for females). 

The amount of calcium in the EAT-IT dietary plan was in line with estimates provided by the EAT-Lancet Commission, who indicated a theoretical value of 718 mg of calcium from the Reference Diet [11]. The differences between the two dietary plans were likely due to the higher amount of milk and cheese included in the IDG compared to the EAT-IT dietary plan. This issue was also highlighted by Lassen et al. [14], who adapted the ELCRD to reach the indications of their Nordic Nutrition Recommendations [30] of 1 g by increasing the portion of dairy foods and cheese. 

However, the calculations performed did not consider the calcium content in water, which could significantly contribute to the intake, reducing the risk of inadequacy with respect to the calcium requirements [31]. The calcium content in tap water may largely vary; thus, it is difficult to evaluate the contribution of water to the total calcium intake [32]. Considering an average value of 60 mg/L for Italian tap waters [33], 2 L provides about 120 mg of highly bioavailable calcium, which still seems to be insufficient to reach the PRI value. Thus, an adequate availability and choice of calcium-rich sources (including mineral waters and/or fortified food products) could be pivotal to avoid the long-term effects of deficiencies of at-risk nutrients, such as calcium, particularly in vulnerable subjects (e.g., women or, in general, subjects with restrictive or less varied eating habits). Moreover, specific guidelines would be fundamental to allow for reaching dietary recommendations depending on the type of source considered.

Regarding vitamin D, both menus were very low (≈2 µg) as sources of this important micronutrient, which has impacts on numerous body functions. These values are in line with the levels of intake according to INRAN-SCAI 2005-06 [29], which indicated a median intake of 1.9 µg for males and 1.5 µg for females in Italy. In addition, the major source for vitamin D is the endogenous synthesis that takes place in the skin, while food sources of vitamin D, such as fatty fish, mushrooms, and eggs, typically play only a minor role in the total contribution [34,35]. However, considering specific members of the population have little or no exposure to sunlight or have a diminished synthesis capacity (e.g., older subjects) [36], the resulting intake of vitamin D represents a problem for which different strategies could be useful, such as the use of well-designed and targeted fortified foods or novel foods [37].

Intriguingly, iron was slightly higher in the EAT-IT dietary plan when compared with the IDG one. This could be explained by considering that, while the amount of red meat was comparable between the two dietary plans, the intake of legumes and nuts was higher in the EAT-IT compared to the IDG. However, iron bioavailability largely depends on the food source and the type of iron. The heme iron present in meat generally shows the highest bioavailability, and, conversely, different types of iron (nonheme) and the co-presence of phytate in plant-based food diets could reduce the bioavailability [38,39]. Conversely, the presence of reducing agents (i.e., vitamin C) should increase the bioavailability [40].

Another apparent difference between the nutritional profile of these two dietary plans is related to sodium and chlorine, which were higher in the IDG dietary plan when compared with the EAT-IT plan. However, in both dietary plans, added salt was not included. The higher amount of sodium and chlorine in the IDG dietary plan could be explained by considering the higher amount of foods containing salt (e.g., bread and cheese) that was suggested by the IDG compared to the EAT-IT. The level of sodium in the IDG dietary plan was slightly above the SDT (without considering added salt) and highlights the importance of policies for reducing the amount of salt contained in foods, which represent the main source of salt (up to 70–75% of the total intake) in Europe [41]. Indeed, the actual intake for the Italian population is acknowledged to be largely higher than recommended by the World Health Organization (WHO) (<5 g day), with an average estimated salt intake of almost 11 g for men and 8.5 for women [42,43]. 

Overall, the comparison between the micronutrients provided by the two dietary plans and dietary recommendations often highlighted that the amount could not always cover the needs of the overall population (i.e., the PRI referring to 97.5% of the population). These findings suggest a potential future need for strategies to improve the nutritional characteristics of foods and diets to enable coverage of the nutrient needs of specific target groups. The strategies may include the formulation of new products that are enriched with specific compounds, for instance, by the selection of new cultivars of vegetable products with increased nutritional benefits or the exploitation of novel foods to define new dietary models that are optimized to cover eventual inadequacies. 

While the simulation of possible dietary patterns with potential benefits in terms of sustainability and human health is a challenging approach with both pros and cons, the present study showed some strengths. For example, the dietary plans were developed by considering the Italian dietary habits, tradition, and culture, as well as in terms of meal composition and distribution throughout the day, with the aim to increase the final acceptability of a future dietary plan. The newly developed dietary pattern was developed to consider several alternatives such that the diet can potentially be used in the medium-to-long term (e.g., months) due to the feasibility of the dietary pattern. 

Regarding limitations, the first was the impossibility of performing an extensive and accurate comparison with dietary recommendations due to missing/unavailable data for certain micronutrients in the food databases, as previously reported. In particular, the micronutrients for which it was not possible to assess nutritional intake included vitamin B5, B8, and K, as well as copper, iodine, manganese, and selenium, which were also not considered in the previously cited Italian National Food Consumption Survey INRAN-SCAI 2005-06 [29]. Even considering other databases, such as the USDA, some micronutrients (i.e., vitamin B8 and iodine) are still not complete. This issue indicates the importance of developing more complete food composition databases to better evaluate the ability of dietary plans to cover nutritional requirements [44]. 

Therefore, future efforts should be focused in this direction to allow for more accurate nutritional assessments, especially considering diets that can be critical for certain nutrients in the overall population or specific target groups [44,45]. Despite this, deficiencies are unlikely for almost all of the abovementioned micronutrients that have incomplete data in the databases, except for iodine, for which almost 45% of the European population showed insufficient intake [21]. These data support the importance of policies that have been established to increase the consumption of iodine-enriched salt and its utilization as an ingredient in food [40]. Second, in the present study, we considered several isoenergetic food alternatives that mimic practical dietary plans that include generally different possible consumer food/meal choices (e.g., whole milk and semi-skimmed milk as alternatives for breakfast) that may differ in nutritional composition. In these cases, we considered the average composition, but it is clear that differences between foods may also impact the final nutrient intake. This further underlines the importance of making dietary choices that are not only focused on energy content but also defining targeted alternatives that are able to cover potential inadequacies. Last, a thorough evaluation of the effect of processing and cooking on the nutrient content was not performed in a systematic way, despite these factors being able to affect the nutritional compositions of dishes.

The recommended portion sizes and frequencies of consumption for the different food groups as proposed in the EAT-IT are quite far from the current dietary behavior of the Italian population. In detail, one of the main differences regards the amount of legumes and nuts that are consumed in a lower amount in the Italian population compared to EAT-IT, while meat, eggs, dairy products, animal fat, and tropical oils are consumed in higher amounts [46].

These wide differences could make it difficult to adhere to the EAT-IT dietary pattern developed; thus, further efforts should be done to enable better compliance and acceptability of the model. Among these efforts, the development of specific food-based dietary guidelines and policies might help consumers with understanding how to tailor their dietary habits in order to achieve a more sustainable dietary pattern that is, at the same time, respectful of local culture and tradition. In this regard, it is worth noting that a new version of the Mediterranean pyramid has recently been proposed, which recommends consuming legumes and nuts every day [47]. The possibility of improving the adoption of eating habits in consumers in line with these guidelines could also be facilitated by implementing attractive recipes and new products that are able to include such foods, and to target different consumer groups, such as the younger consumers who are very often those that are less adherent to the Mediterranean and healthy dietary patterns. In this scenario, the availability of fortified products or alternative sources may become a good option above all in those consumers who are less prone to adapting to new dietary patterns and foods or more strict guidelines.

## 5. Conclusions

Overall, the newly developed dietary pattern represents a possible practical elaboration of the data indicated by Willet et al. [11], but it should not be considered as strict or prescriptive. The defined dietary pattern is intended for adult subjects having a 2500 kcal daily energy need, but adjustments for other energy targets or population groups (e.g., older adults) should be made. Therefore, further efforts are needed to define the concrete feasibility of such patterns in relation to the food habits of the population, considering that making changes that are too large from their usual diet can preclude many people from adopting such diets [10]. Finally, there is a clear need for the validation of these newly developed dietary patterns within the context of proper real-life studies to better elucidate their feasibility, affordability, and their beneficial health effects.

## Figures and Tables

**Table 1:** Reference scheme for breakfast and snacks.

**(1A) Breakfast**		
**Food Category**	**Portion Size**	**Possible Alternatives**
(a) Dairy	155 kcal	250 mL whole milk 330 mL semi-skimmed milk 230 g whole milk yogurt 350 g semi-skimmed yogurt 20 g butter
(b) Cereal	170 kcal	45 g cornflakes 45 g rusks (5 slices) 75 g whole bread
(c) Sugars *	120 kcal	50 g jam 220 mL fruit juice
**(1B) Snack**		
**Food Category**	**Portion Size**	**Practical Examples (Edible Part)**
Nuts	290 kcal	Almonds—50 g Peanuts—50 g Pistachios—50 g Walnuts—40 g

Breakfast (1A) is composed of a portion of (a) dairy, (b) cereals, and (c) sugars. Snacks (1B) are composed of a portion of nuts. The amount of nuts indicated in the 1B scheme for snacks can be eaten as a single snack or on two occasions per day, halving the total amount indicated in the table for each occasion. * The EAT-Lancet Commission reference diet indicates 25 g of sugars per day, which was considered as “free sugars” that is “all monosaccharides and disaccharides added to foods by the manufacturer, cook or consumer, plus sugars naturally present in honey, syrups, and fruit juices,” as indicated by the World Health Organization (WHO) [22].

**Table 2:** Reference scheme for meals.

**(a) Whole Grains**				
**Lunch (400 kcal)**		**Dinner (275 kcal)**		
**Food**	**Theoretical Portion**	**Food**	**Theoretical Portion**	
Brown rice (364 kcal/100 g)	120 g	Brown rice (364 kcal/100 g)	80 g	
Spelt (335 kcal/100 g)	120 g	Spelt (335 kcal/100 g)	80 g	
Whole pasta (355 kcal/100 g)	120 g	Whole pasta (355 kcal/100 g)	85 g	
Corn (365 kcal/100 g)	110 g	Corn (365 kcal/100 g)	80 g	
Common bread (275 kcal/100 g)	180 g	Common bread (275 kcal/100 g)	120 g	
		Potatoes (78 kcal/100 g)	325 g	
**(b) Protein Sources**				
**Food**	**Energy to Create Harmonized Portion**	**Theoretical Average Energetic Density**	**Weekly Consumption**	**Theoretical Portion**
Beef, lamb, and pork	215 kcal	214 kcal/100 g	1 time	100 g
Chicken and other poultry	215 kcal	214 kcal/100 g	2 times	100 g
Eggs	160 kcal	146 kcal/100 g	1 time (2 eggs)	125 g
Fish	150 kcal	143 kcal/100 g	2 times	105 g
Legumes (dried)	245 kcal	379 kcal/100 g	8 times	65 g
**Others**				
(c) Vegetables	Approximately 40 kcal (150 g/meal)			
(d) Oil and seasoning	Approximately 25 g/meal mainly from food sources rich in			
	unsaturated fatty acids, such as extra-virgin olive oil			
(e) Fruit	Approximately 60 kcal (100 g/meal)			

Meals (lunch and dinner are composed of a portion of (a) whole grains, (b) protein sources, (c) vegetables, (d) oil and seasoning, and (e) fruit. The main meals, namely, lunch and dinner, include (i) a portion of cereals or potatoes, (ii) a portion of a protein source in relation to their calculated frequencies of consumption, (iii) a portion of vegetables, (iv) oil and seasoning, and (v) a portion of fruit to end the meal. This table has no prescriptive value; thus, lunch and dinner can be interchanged. This table can be theoretically used to compose complete dishes (e.g., “pasta al ragù” + fruit) or a meal composed of a single portion of each component (omelet with spinach, whole bread, and fruit).

**Table 3:** Comparison between the suggested portions in the Italian dietary guidelines for healthy eating (for a 2500 kcal diet) and the EAT-IT dietary plan (i.e., the ELCRD tailored to consider Italian food habits), which was developed based on the planetary healthy diet.

	Italian Guidelines		EAT-IT Dietary Pattern
Food Group	Food Subcategory	Daily or Weekly Portion	
Cereals and derivatives	Bread	4.5 portions/day of 50 g (225 g/day)	≠ Max daily amount of whole grain bread of about 375 g
	Pasta, rice, corn, spelt, and barley	1.5 portions/day of 80 g (120 g/day)	≠ Max daily amount of about 200 g
	* Bread substitutes (rusks, crackers, and breadsticks)	1 portion/week of 30 g (30 g/week)	≠ About 45 g of rusks (five slices) can be eaten at breakfast
	* Sweet bakery products (brioche, croissants, and biscuits)	2 portions/week of 50 g for croissants or cake or 30 g/week for biscuits (100 or 60 g/week)	≠ Sweet products can be eaten at breakfast and are indicated as “sugars and other sweeteners”
	* Breakfast cereals	2 portions/week of 30 g (60 g/week)	≠ About 45 g of breakfast cereals can be eaten at breakfast
Tubers	Potatoes	2 portions/week of 200 g (400 g/week)	↓ 1 portion/week of 325 g (325 g/week)
× Fruits	Fresh fruits	3 portions/day of 150 g (450 g/day)	↓ 200 g/day
	Dried fruits	3 portions/day of 30 g (90 g/day)	n.s.
× Vegetables	Fresh vegetables	3 portions/day of 200 g (600 g/day)	↓ 300 g/day
	Leaf salad	3 portions/day of 80 g (240 g/day)	n.s.
Meat	* Red meat (beef, pork, and sheep meat)	1 portion/week of 100 g (100 g/week)	Beef, lamb, or pork—100 g/week (100 g/week)
	White meat (chicken, turkey, or rabbit)	3 portions/week of 100 g (300 g/week)	↓ Chicken and other poultry—2 portions of 100 g/week (200 g/week)
Fishery	Fish (including mollusks and crustaceans)	3 portions/week of 150 g (450 g/week)	↓ Fish—2 portions/week of 105 g (210 g/week)
	* Preserved fish (e.g., canned tuna)	1 portion/week of 50 g (50 g/week)	n.s.
Egg	Egg	4 medium eggs/week (200 g/week)	↓ 1 portion/week of 2 medium eggs (125 g/week)
× Legumes	Fresh legumes or canned	3 portions/week of 150 g (450 g/week)	↑ 8 portions/week of 65 g of dried legumes—about 200 g of fresh legumes (520 g or 1560 g/week)
	Dried legumes	3 portions/week of 50 g (150 g/week)	
× Milk and derivatives	Milk	3 portions/day of 125 mL (375 mL/day)	↓ 1 portion/day of 250 mL of milk or other isocaloric equivalences of milk derivatives (e.g., yogurt, butter, etc.) (250 mL/day)
	Yogurt and other fermented milk	3 portions/day of 125 g (375 mL/day)	
	Cheese (fat <25% and less than 300 kcal/100 g)	3 portions/week of 100 g (300 g/week)	
	Cheese (fat >25% and more than 300 kcal/100 g)	3 portions/week of 50 g (150 g/week)	
× Fats and seasoning	Vegetable oil (e.g., extra virgin olive oil and seed oil)	4 portions/day of 10 mL (40 mL/day)	↑ 50 g/day of added fats, preferably from dietary plant sources. Butter is excluded because it is already included in the milk and derivatives food category
	Butter and other animal fats	4 portions/day of 10 g (40 g/day)	
Nuts and seed	Walnuts, peanuts, almonds, seeds, etc.	2.5 portions/week of 30 g (75 g/week)	↑ 40–50 g/day
Water	Water	At least 10 glasses of 200 mL/day (2 L/day)	n.s.

n.s.: not specified. ×: the portions reported for the food included in that category are alternatives and not additive (e.g., for “fruits,” 150 g of fresh fruit OR 30 g of dried fruit); *: subcategory for which it is possible to have a lower frequency of consumption and increasing the consumption of other foods from the same category, according to the Italian dietary guidelines (IDG). ≠: food category with different recommendations between the IDG and EAT-IT but not clearly definable in terms of whether the amount is higher, equal, or lower. ↑↓ higher or lower recommendations, respectively, in the EAT-IT dietary pattern compared to the IDG.

**Table 4:** Comparison between the macronutrients provided by the IDG and EAT-IT dietary plans for a 2500 kcal diet.

Macronutrient Intake				
Nutrient	IDG	EAT-IT		LARN (Adults)
Energy	2500	2500	kcal	
Protein	97.7	97.6	g	AR 0.71 g/kg × die (PRI 0.9 g/kg × die)
Energy protein/total energy	15.6	15.6	%	12–18% En †
Animal protein	47.6	35.6	g	
Animal protein/total protein	48.7	36.5	%	
Vegetal protein	50.2	62.0	g	
Vegetal protein/total protein	51.3	63.5	%	
Lipids	84.3	100.6	g	
Energy lipids/total energy	30.3	36.2 *	%	RI 20–35% En
SFA	23.2	19.3	g	
Energy SFA/total energy	8.4	7.0	%	SDT < 10% En
MUFA	41.3	54.5	g	
Energy MUFA/total energy	14.9	19.6	%	
PUFA	10.3	17.4	g	
Energy PUFA/total energy	3.7 *	6.3	%	RI 5–10% En
Total ω-6	8.7	15.0	g	
Energy ω-6/total energy	3.1 *	5.4	%	RI 4–8% En
Total ω-3	1.8	2.1	g	
Energy ω-3/total energy	0.6	0.8	%	RI 0.5–2.0% En
Cholesterol	248.7	165.4	mg	SDT < 300 mg
Carbohydrates	317.3	276.8	g	
Energy carbohydrates/total energy ¥	53.9	47.8	%	RI 45–60% En
Sugars ×	111.5	85.3	g	
Energy sugars/total energy	17.8 *	13.6	%	SDT < 15% En
Total fiber	39.1	44.1	g	SDT > 25 g/die
Total fiber/1000 kcal	15.6	17.7 *	g	RI 12.6–16.7 g/1000 kcal
Energy total fiber/total energy	3.1	3.5	%	

AR: average requirement; EAT-IT: dietary pattern based on the EAT-Lancet Commission Reference Diet with adaptations for the Italian population; En: energy; IDG: Italian Dietary Guidelines; LARN: Reference Intake of Nutrients and Energy for the Italian Population; MUFA: monounsaturated fatty acids; PRI: population reference intake; RI: reference intake; SDT: standard dietary target; SFA: saturated fatty acids; PUFA: polyunsaturated fatty acids. *: Deviations from the reference requirements; †: range of energy from protein considered as an acceptable level of consumption (not an RI itself) in the LARN; ¥: energy from carbohydrates includes energy from fiber; ×: sugars contained in foods, including added sugars, sugars naturally occurring in milk, fruit, and vegetables, as reported in the LARN.

**Table 5:** Comparison between the vitamins provided by the IDG and EAT-IT dietary plans for a 2500 kcal diet.

Vitamin (Vit.) Intake					
Nutrient	IDG	EAT-IT		LARN (Adults 18–59 Years)	
				AR	PRI or AI ^§^
Vit. A (retinol eq.)	2400	1500	µg	Male 500 µg (female 0.4 mg)	PRI male 700 µg (female 600 µg)
Vit. D (cholecalciferol, ergocalciferol)	2.3 *	1.9 *	µg	10 µg	PRI 15 µg
Vit. E (tocopherols, tocotrienols)	17.1	21.6	mg		AI male 13 mg (female 12 mg)
Vit. B1 (thiamine)	1.4	2.5	mg	Male 1 mg (female 0.9 mg)	PRI male 1.2 mg (female 1.1 mg)
Vit. B2 (riboflavin)	2.4	1.6	mg	Male 1.3 mg (female 1.1 mg)	PRI male 1.6 mg (female 1.3 mg)
Vit. B3 (niacin)	23.0	26.0	mg	14 mg	PRI 18 mg
Vit. B6 (pyridoxine)	2.8	3.2	mg	1.1 mg	PRI 1.3 mg
Vit. B9 (folic acid)	617.5	433.7	µg	320 µg	PRI 400 µg
Vit. B12 (cyanocobalamin)	4.3	3.3	µg	2 µg	PRI 2.4 µg
Vit. C (ascorbic acid)	250.9	175.5	mg	Male 75 mg (female 60 mg)	PRI male 105 mg (female 85 mg)

IDG: Italian Dietary Guidelines; EAT-IT: dietary pattern based on the EAT-Lancet Commission Reference Diet with adaptations for the Italian population; LARN: Reference Intake Levels of Nutrients and Energy for the Italian Population; AR: average requirement; PRI: population reference intake; AI: adequate intake; ^§^: AI was obtained from the average intakes observed in the apparently healthy population free from deficiencies. It was used as a substitute for AR and PRI when these indicators could not be formulated based on available scientific evidence. *: The level of intake for the respective nutrient was inadequate to satisfy the nutritional requirements.

**Table 6:** Comparison between minerals provided by the IDG and EAT-IT dietary plans for a 2500 kcal diet.

Mineral Intake						
Nutrient	IDG	EAT-IT		LARN (Adults 18–59 Years)		
				AR	PRI or AI ^§^	SDT
Calcium	1079.1	675.6 *	mg	800 mg	PRI 1000 mg	
Sodium	2070.3 *	826.9	mg		AI 1500 mg	<2000 mg
Chlorine	1217.0	531.0	mg		AI 2300 mg	<3000 mg
Iron	17.9	22.1	mg	Male 7 mg (female 10 mg)	PRI male 10 mg (female 18 mg)	
Magnesium	356.2	491.4	mg	170 mg	PRI 240 mg	
Phosphorus	1851.4	1867.0	mg	580 mg	PRI 700 mg	
Potassium	4939.2	4609.5	mg		AI 3900 mg	
Zinc	14.8	15.9	mg	Male 10 mg (female 8 mg)	PRI male 12 mg (female 9 mg)	

AI: adequate intake; AR: average requirement; EAT-IT: dietary pattern based on the EAT-Lancet Commission Reference Diet with adaptations for the Italian population; IDG: Italian Dietary Guidelines; LARN: Reference Intake Levels of Nutrients and Energy for the Italian Population; PRI: population reference intake. ^§^: AI was obtained from the average intakes observed in an apparently healthy population free from manifest deficiencies. It was used as a substitute for AR and PRI when these indicators could not be calculated based on available scientific evidence. *: The level of intake for the respective nutrient was inadequate to satisfy the nutritional requirements.

## Appendix A

Glossary of the main terms used.

**Name**	**Definition**
ELCRD (EAT-Lancet Commission Reference Diet)	Proposal included in a recent report from the EAT-Lancet Commission [11], consisting of indications of consumption for different food categories within which it is possible to have a simultaneously sustainable and healthy diet. This set of indications can be theoretically adapted to all food contexts, and for this reason, is sometimes indicated as a “planetary diet”
EAT-IT dietary pattern	Proposal for a local adaptation of the ELCRD to the Italian/Mediterranean food context, which is organized in a schematic quali-quantitative representation that is feasible for mid/long-term utilization
EAT-IT-based dietary plan	Simulation of the application of the EAT-IT dietary pattern in which a long-term menu made of recipes and dishes that are consistent with EAT-IT was designed
IDG (Italian Dietary Guidelines) [18]	Official national dietary guidelines for the Italian population and was recently revised in their latest edition (2018)
IDG based dietary plan	Simulation of a long-term menu that is made of recipes and dishes that are consistent with the indications provided by the IDG
LARN (Reference Intake of Nutrients and Energy for the Italian Population) [21]	Consensus document that includes reference values for the intakes of nutrients and energy to cover the nutritional requirements of different target groups in the Italian population
